# Spatial and temporal evolution characteristics of apple carbon emissions in China, 1991–2020

**DOI:** 10.1038/s41598-025-02419-8

**Published:** 2025-05-27

**Authors:** Qiangqiang Zhang, Xixi Gao, Yujie Hu, Zhongwei He, Xiaojing Li

**Affiliations:** 1 Beijing Research Center for Rural Revitalization/College of Economics and Management, Beijing University of Agriculture, Beijing, 102206 China; 2 Faculty of Applied Economics, University of Chinese Academy of Social Sciences, Beijing, 102488 China; 3 School of Business, University of Leeds, Leeds, UK; 4 School of Economics and Management, Yantai University, Yantai, 264005 China

**Keywords:** Apple carbon emissions, Temporal characteristics, Regional differences, Spatial effects, China, Environmental impact, Environmental impact

## Abstract

Articulating the spatial and temporal features of apple carbon emissions in China across different years and areas is critical for developing an appropriate and acceptable apple carbon reduction program. This paper builds a provincial-level database (1991–2020) of apple carbon emissions in China, covering six primary sources: chemical fertilizers, pesticides, plastic films, machinery, irrigation, and tillage. By using the LMDI approach, spatial autocorrelation analysis, and barycenter analysis, we find that (1) China’s apple carbon emissions exhibited an M-shaped fluctuation pattern, with a 1.38-fold increase during the study period, while apple carbon intensity increased 67.01%. (2) Chemical fertilizers were the largest contributor (55%) to apple carbon emissions, followed by plastic films, irrigation, pesticides, machinery, and tillage. (3) Apple carbon emissions showed significant regional heterogeneity, with a spatial pattern of the Bohai Bay production area as the largest in terms of carbon emissions and carbon intensity. While chemical fertilizers were the primary contributor in the other four production areas, plastic films had a disproportionately high impact in Bohai Bay production area. Moreover, 13 provinces increased their carbon emissions, while 9 provinces reduced. Chemical fertilizers, plastic films and irrigation were dominant contributing factors in 14, 5, and 3 provinces, respectively. (4) There is no significant spatial association between surrounding provinces in apple carbon emissions. Spatial correlations reveal a “high-low” or “low-high” agglomeration of negative values during certain individual years. In 2020, the barycenter of apple carbon emissions migrated southwestward 451.69 km, reaching Shaanxi Province. This article recommends promoting organic fertilizers, water-fertilizer integration, mulch recycling, straw mulching, and drip irrigation to minimize apple carbon emissions. Additionally, region-specific emission reduction strategies should be developed, with particular emphasis on Shaanxi and Gansu, considering their distinct spatial and temporal characteristics.

## Introduction

Global warming poses serious threats, including glacier melting, sea-level rise, freshwater depletion, and ecological degradation, all of which endanger human survival and development^1,2^. Carbon emissions are a primary greenhouse gases (GHG) that contribute to global warming, and as a result, many governments worldwide are prioritizing carbon emission reductions as a key strategy to addressing this pressing issue^3,4^. The seventh measure towards attaining carbon peak emphasizes the reduction and storage of carbon emissions from agriculture and rural regions^5^, which necessitates a rapid decrease in carbon emissions from agricultural production operations^6^. Agricultural production, including activities such as land use, crop cultivation, and animal husbandry, is the second largest source of greenhouse gas emissions^7^, all of which contributing significantly to global warming^8,9^. According to the Intergovernmental Panel on Climate Change (IPCC) Sixth Assessment Report (AR6), agricultural, forestry, and other land use (AFOLU) activities accounted for 13-21% of global GHG emissions between 2010 and 2019^10,11^.

China is the world’s greatest emitter of carbon dioxide (CO_2_), accounting for 30.64% of worldwide emissions in 2020^12–14^. China, as a major agricultural country, employs approximately 177 million people in agriculture, representing 2.28% of the world population in 2020^15^. The agricultural sector is a large source of the country’s carbon emissions^16^, accounting for 17% of total national carbon emissions^17,18^. Meanwhile, urbanization and industrialization have increased the movement of rural labor to urban areas. The percentage of agricultural labor decreased from 70.5% in 1978 to 23.6% in 2020^19^. To maintain agricultural production and ensure a stable supply of agricultural products, the use of capital-intensive inputs such as fertilizers, pesticides, agricultural films, and machinery has increased^6^, adding further pressure on China to reduce agricultural carbon emissions^20,21^.

Numerous studies have investigated various aspects of agricultural carbon emissions in China, including their measurement^6,13,22^, characteristics^23–25^, influencing factors^26–28^ and effects^29–31^. For example, Shan et al. estimated China’s agricultural carbon emissions across 30 provinces for the period 1997–2015 using the accounting method for carbon emissions devised by the IPCC^13^. Chen et al. analyzed the spatial-temporal evolution characteristics of agricultural carbon emissions in Fujian Province from 2008 to 2017, identifying a fluctuating downward trend in total emissions, with inland areas producing more emissions than those from the eastern coastal areas^23^. Sui & Lv indicated that the drivers of agricultural carbon emissions can be decomposed into emission intensity, structure, economic factors and labor effect, with economic growth in agriculture being the main driver^27^. Luo et al. found that between 1997 and 2014, East China saw a strong decoupling of CO_2_ emissions from agricultural output value in several periods^29^.

Despite extensive research on China’s agricultural carbon emissions, little attention has been given to emissions from apple production. China is the largest apple producer in the world, with 45.0 million tons produced in the 2021/22 season^32,33^. Fruit trees are an important component of China’s agriculture, with 12.65 million hectares of orchards, including 1.99 million hectares of apple orchards in 2020^19^. China’s apple orchards significantly contribute to the carbon emissions of terrestrial ecosystems due to their planted area and emissions capacity. However, there is limited understanding of the carbon emissions associated with China’s apple orchards.

To address this gap, this paper constructs a database of apple carbon emissions at the national, regional and provincial levels in China from 1991 to 2020. The database covers the carbon emissions of chemical fertilizers, pesticides, plastic films, machinery, irrigation and tillage during apple cultivation. The contributions of this paper are as follows. First, unlike most studies that focus on agricultural carbon emissions at a macro level, this research specially investigates apple production, a relatively underexplored crop. Second, the logarithmic mean Divisia index (LMDI) approach is applied to study the main drivers of changes in apple carbon emissions, providing a reference for establishing effective apple carbon emission reduction mechanisms and informing local sustainable development policies at the provincial level in China. Third, exploratory spatial data analysis (ESDA) is used to evaluate the spatial and temporal evolution of apple carbon emissions at the provincial level in China during the period of 1991–2020. Distinguishing from the simple arithmetic statistical descriptions in existing studies, this paper uses spatial correlation and gravity analysis methods to account for the geospatial distribution of apple carbon emissions over time, so as to realize a dynamic and comprehensive understanding of the panel data. The main objectives of this paper are to investigate the spatial and temporal evolution of carbon emissions from apples in China from 1991 to 2020 and to provide a reference for the next step in reducing apple-related carbon emissions.

The remaining structure of the article is as follows. Materials and methods are described in Sect. 2. Section 3 presents the empirical results, including the LMDI decomposition of the drivers of apple carbon emissions and the spatial-temporal characteristics of emissions at the provincial level during the study period. Section 4 provides the conclusions, policy implications, and limitations.

## Materials and methods

### Data sources

In this paper, apples are used as the research object, focusing on 23 provinces in China from 1991 to 2020. The selected provinces include Beijing, Tianjin, Hebei, Liaoning, Shandong, Shanxi, Shaanxi, Gansu, Qinghai, Ningxia, Jiangsu, Anhui, Henan, Sichuan, Chongqing, Guizhou, Yunnan, Tibet, Inner Mongolia, Jilin, Heilongjiang, Hubei and Xinjiang. Eight non-apple producing provinces were excluded from the analysis. Apple carbon emissions data were derived from the China Rural Statistical Yearbook (1992–2021) and provincial yearbooks, including information on chemical fertilizers application for agricultural use (in discounted pure form), pesticides, agricultural plastic films, total power of agricultural machinery, irrigated arable land area, actual apple orchard area, and total sown area of crops.

Noteworthy, data on chemical fertilizers, pesticides, agricultural film, agricultural machinery, irrigation and tillage related to apple production are not directly available in Chinese statistical records, so this paper uses the processing methods of Feng et al.^34^ to pre-process the data. Tillage data were replaced by the area of apples planted in the corresponding year. The proportion of apple planted area to the total sown area of crops was multiplied by the original data of each indicator in the yearbooks to estimate apple-related inputs. For example, to estimate apple-related chemical fertilizers input, the proportion of apple planted area to total crop sown area was multiplied by the number of agricultural chemical fertilizer application rates recorded in the yearbooks. The same method was applied to estimate pesticide use, plastic film application, total agricultural machinery power, and irrigation. This method has advantages in terms of data availability and operational simplicity but has limitations in terms of reduced data accuracy the inability to account for differences in input use and regional variation. The converted data can only provide a rough estimate.

As Chongqing was established as a municipality in 1997, its data was combined with Sichuan Province in this paper to maintain data consistency and comparability^32^. After this adjustment, the number of apple-producing provinces analyzed in this paper was reduced to 22. Missing data were filled using the mean interpolation method, which provides reasonable estimates for missing values caused by random factors^35^. The map data were sourced from the National Centre for Basic Geographic Information (www.webmap.cn.), with vector data at a scale of 1:1 million and a is Krasovsky-1940-Albers geographic coordinate system.

According to the apple industry layout plan designated by the Ministry of Agriculture and Rural Affairs of People’s Republic of China, China’s apple production areas are grouped into five regions: the Bohai Bay production area (BBPA) (including Beijing, Tianjin, Hebei, Liaoning and Shandong), the Loess Plateau production area (LPPA) (including Shanxi, Shaanxi, Gansu, Qinghai and Ningxia), the Yellow River Old Road production area (YRORPA) (including Jiangsu, Anhui and Henan), the Southwest Cold and Highland production area (SCHPA) (including Sichuan, Chongqing, Guizhou, Yunnan and Tibet) and the Special production area (SPA) (including Inner Mongolia, Jilin, Heilongjiang, Hubei and Xinjiang) . Among these regions, the BBPA and LPPA are two dominant apple production areas in China^32,36^.

### Calculation method of apple carbon emissions

Carbon dioxide emissions are generated through the use of agricultural chemical fertilizers, pesticides, plastic films, machinery, irrigation and tillage, in apple orchard production^17,37^. Some CO_2_ is released to the atmosphere in the production of apple orchard. Drawing on existing studies, this paper identifies six primary sources of carbon emissions associated with apple land use: chemical fertilizers, pesticides, agricultural films (such as black mulch film (using for soil moisture retention), reflective film (using for reflecting sunlight to promote fruit reddening), agricultural machinery (such as rototillers, sprayers, mowers, tractors, tricycle), irrigation, tillage, which directly or indirectly caused carbon emissions^9,34^. Following the methodology outlined by the IPCC^38^, apple carbon emissions are calculated through Eq. (1):

1 $$\:{E}_{k,t}={\sum\:}_{i=1}^{6}{E}_{k,t,i}={\sum\:}_{i=1}^{6}\left({T}_{k,\:t,i}{\cdot\:\mu\:}_{i}\right)$$

where $\:{E}_{k,t}$ denotes the apple carbon emissions amount of the$\:k$ province in $\:t$ year ($\:k=1,\:\:\dots\:,\:22$. $\:t=1991,\:\:\dots\:,\:2020$). $\:{E}_{k,t,i}$ represents the actual carbon emissions amount of carbon emission source$\:i$ (including chemical fertilizers, pesticides, agricultural films, agricultural machinery, irrigation, tillage) of the $\:k$ province in $\:t$ year. $\:{T}_{k,t,i}$ means the actual amount of carbon emission source $\:i$ of the $\:k$ province in $\:t$ year. $\:{\mu\:}_{i}$ is the carbon emission coefficient of source $\:i$. The carbon emission coefficients corresponding to each source are listed in (Table 1).

**Table 1:** Carbon emission coefficients for each source.

Carbon source	Emission coefficient	Unit	Data reference source
Chemical fertilizers	0.8956	Kg CE/kg	ORNL^①^, Tian et al.^39^
Pesticide	4.9341	Kg CE/kg	ORNL^①^, Li et al.^40^
Plastic film	5.1800	Kg CE/kg	IREEA^②^, Tian et al.^39^
Machinery	0.1800	Kg CE/kw	Zhu et al.^41^
Irrigation	266.4800	Kg CE/hm^2^	West & Marland^42^
Tillage	312.6000	Kg CE/km^2^	CABCAU^③^, Wu et al.^43^

① ORNL denotes Oak Ridge National Laboratory of America: https://www.ornl.gov/. ② IREEA represents Institute of Resource, Ecosystem and Environment of Agriculture: https://ireea.njau.edu.cn/. ③ CABCAU refers to College of Agronomy and Biotechnology, China Agricultural University: http://cbs.cau.edu.cn/sys/.

### Calculation method of carbon emission contribution from each source

Changes in inputs, such as chemical fertilizers, pesticides, plastic films, machinery, irrigation and tillage, drive increases or decreases in apple carbon emissions. To compare the contribution of each carbon source to the change of apple carbon emissions, this paper uses the logarithmic mean Divisia index (LMDI) method. This approach decomposes the changes in apple carbon emissions within a given region into the incremental contributions of six carbon emission sources: chemical fertilizers input (referred to as fertilizer contribution), pesticide input (referred to as pesticide contribution), plastic films input (referred to as plastic films contribution), farm machinery input (referred to as machinery contribution), irrigation acreage (referred to as irrigation contribution) and tillage acreage (referred to as tillage contribution), that is, the change in apple carbon emissions can be expressed as Eq. (2):

2 $$\:\varDelta\:{E}_{r,c}={\sum\:}_{i=1}^{6}\varDelta\:{E}_{r,c,i}={\sum\:}_{i=1}^{6}\left(\frac{{E}_{r,c}-{E}_{r,b}}{{ln}{E}_{r,c}-{ln}{E}_{r,b}}{\:\cdot\:\:ln}\frac{{T}_{r,c,i}}{{T}_{r,b,i}}\right)$$

where $\:\varDelta\:{E}_{r,c}$ denotes the change in apple carbon emissions amount of the $\:r$ region in$\:c$ year (current-period is $\:c$ and $\:c=1992,\:\:\dots\:,\:2020$). $\:\varDelta\:{E}_{r,c,i}$ represents the incremental amount of apple carbon emissions contributed by source $\:i$ of the $\:r$ region in $\:c$ year. $\:{E}_{r,c}$ and $\:{E}_{r,b}$ are the apple carbon emissions amount of the $\:r$ region in$\:c$ year and $\:b$ year (base-period is 1991), respectively. $\:{T}_{r,t,i}$ and $\:{T}_{r,b,i}$ refer to the actual amount of carbon emission sources $\:i$ of the region $\:r$ in $\:c$ year and $\:b$ year, respectively.

The contribution proportion of each carbon source to the incremental apple carbon emissions is calculated through formula (3):

3 $$\:{P}_{r,c,i}=\frac{\varDelta\:{E}_{r,c,i}}{\varDelta\:{E}_{r,c}}\:\cdot\:100\%$$

where $\:{\text{P}}_{\text{r},\text{c},\text{i}}$ denotes the contribution proportion of carbon source $\:\text{i}$ to incremental apple carbon emissions of the $\:\text{r}$ region in $\:\text{c}$ year, and $\:{\sum\:}_{\text{i}=1}^{6}{\text{P}}_{\text{r},\text{c},\text{i}}=100\text{\%}$. In this paper, based on the classification method of Zhang et al.^36^, the contribution proportion can be classified as more significant [50% < max($\:{\text{P}}_{\text{r},\text{c},\text{i}}$) ≤ 60%], significant [60% < max($\:{\text{P}}_{\text{r},\text{c},\text{i}}$) ≤ 80%] and extremely significant [max($\:{\text{P}}_{\text{r},\text{c},\text{i}}$) > 80%] in that order.

### Calculation method of the spatial characteristics of apple carbon emissions

#### Spatial autocorrelation analysis

Spatial autocorrelation analysis is a key component of exploratory spatial data analysis, used to analyze the spatial correlation characteristics of the same attribute variable within a certain region, which includes global spatial autocorrelation and local spatial autocorrelation. The global spatial autocorrelation method analyzes the correlation characteristics and degree of variation of an attribute between different spatial observations by calculating spatial autocorrelation index^37^. Among various global spatial autocorrelation indices (such as Global Geary’s and Gettis’ G), the Global Moran’s I index is widely employed, due to its independence from the assumption of a normal distribution^44,45^. This paper applied the Global Moran’s I index to test whether apple carbon emissions within a region are significantly correlated with those in neighboring regions. The Global Moran’s I index is calculated as shown in formula (4):

4 $$\:Global\:Moran{\prime\:}s\:I=\frac{1}{{\sum\:}_{p=1}^{22}{\sum\:}_{q=1}^{22}{w}_{pq}}\cdot\:\frac{{\sum\:}_{p=1}^{22}{\sum\:}_{q=1}^{22}{w}_{pq}\left({E}_{p,t}-{\stackrel{-}{E}}_{t}\right)\left({E}_{q,t}-{\stackrel{-}{E}}_{t}\right)}{{{\sum\:}_{p=1}^{22}\left({E}_{p,t}-{\stackrel{-}{E}}_{t}\right)}^{2}/22}$$

where $\:{E}_{p,t}$ and $\:{E}_{q,t}$ denote the actual carbon emissions of the province $\:p$ and province $\:q$ in $\:t$ year, respectively. $\:{\stackrel{-}{E}}_{t}$ is the average value of apple carbon emissions for all provinces in $\:t$ year. $\:{w}_{pq}$ indicates the spatial weights between provinces $\:p$ and $\:q$ using Rook adjacency, that is, when province $\:p$ and province $\:q$ have a common edge, $\:{w}_{pq}=1$; otherwise, $\:{w}_{pq}=0$. The *Global Moran’s I* index takes values between − 1 and 1, and if its value is less than 0, it means that a negative agglomeration of regional apple carbon emissions. Otherwise, there is a positive agglomeration. The closer the value is to 0, the weaker the space autocorrelation of apple carbon emissions is. A zero value for *Global Moran’s I* index indicates that there is no spatial autocorrelation of apple carbon emissions across the region, that is, it is spatially distributed randomly over the whole area.

Local spatial autocorrelation allows the exploration of spatial information, such as the location of observations clustered in space, thus providing a comprehensive picture of apple carbon emission trends in regional spatial variation. Its calculation formula is shown in Eq. (5):

5 $$\:Local\:Moran{\prime\:}s\:I=\frac{22\left({E}_{p,t}-{\stackrel{-}{E}}_{t}\right){\sum\:}_{q=1}^{22}{w}_{pq}\left({E}_{q,t}-{\stackrel{-}{E}}_{t}\right)}{{\sum\:}_{p=1}^{22}{\left({E}_{p,t}-{\stackrel{-}{E}}_{t}\right)}^{2}}$$

where $\:{E}_{p,t}$, $\:{E}_{q,t}$, $\:{\stackrel{-}{E}}_{t}$, and $\:{w}_{pq}$ have the same meaning as in formula (4). If the *Local Moran’s I* index is greater than 0, it means that the apple carbon emission of province $\:p$ and its adjacent provinces $\:q$ are all high emissions areas or low emissions areas, that is, the presence of high-high (H-H) or low-low (L-L) spatial agglomerations in the local regions. A negative value indicates the presence of high-low (H-L) or low-high (L-H) spatial agglomerations in the local regions. That is, a high-emission province is surrounded by low-emission provinces, or a low-emission province is surrounded by high-emission provinces. The higher the absolute value of the *Local Moran’s I* index, the higher the degree of spatial agglomeration in the local regions.

#### Spatial barycenter analysis

With the development of 3 S technology, the barycenter model has emerged as an innovative tool for spatially and dynamically examining regional disparities^46–48^. Borrowed from physics, the concept of barycenter in geography represents a point in regional space where forces in all directions remain relatively balanced^49,50^. This paper uses the barycenter model to analyze shifts in the distribution of apple carbon emissions in the sample period. We assume that the provincial region is a homogeneous plane and that the coordinates of its provincial centroid are those of the geographical barycenter^36^. Thus, the barycenter of apple carbon emissions is the equilibrium point in the provincial distribution of apple carbon emissions in a given year, it can be calculated using formula (6).

6 $$\:{\overline{X}}_{t}={\sum\:}_{k=1}^{22}\frac{{E}_{kt}{X}_{k}}{{\sum\:}_{k=1}^{22}{E}_{kt}};\:{\overline{Y}}_{t}={\sum\:}_{k=1}^{22}\frac{{E}_{kt}{Y}_{k}}{{\sum\:}_{k=1}^{22}{E}_{kt}}$$

where $\:{\overline{X}}_{t}$ and $\:{\overline{Y}}_{t}$ denote the longitude and latitude of apple carbon emission barycenter in $\:t$ year, respectively. $\:{X}_{k}$ and $\:{Y}_{k}$ is the longitude and latitude of geographical barycenter of the $\:k$ province, respectively. $\:{E}_{kt}$ indicates the apple carbon emissions of the $\:k$ province in $\:t$ year.

According to the coordinates of barycenter, the moving distance of the apple carbon emission barycenter can be calculated through formula (7):

7 $$\:{D}_{T-t}=\phi\:\times\:\sqrt{{\left({\overline{X}}_{T}-{\overline{X}}_{t}\right)}^{2}+{\left({\overline{Y}}_{T}-{\overline{Y}}_{t}\right)}^{2}}$$

where $\:{\text{D}}_{\text{T}-\text{t}}$ indicates the moving distance of China’s apple carbon emission barycenter from $\:\text{t}$ year to $\:\text{T}$ year. $\:\left({\overline{\text{X}}}_{\text{t}},{\overline{\text{Y}}}_{\text{t}}\right)\:$and $\:\left({\overline{\text{X}}}_{\text{T}},{\overline{\text{Y}}}_{\text{T}}\right)\:$are the latitude and longitude coordinates of China’s apple carbon emission barycenter in $\:\text{t}$ year and $\:\text{T}$ year, respectively. $\:{\upphi\:}$ represents a planar geographic distance conversion factor, which can transfer China’s apple carbon emission barycenter from geographic coordinates to a plane distance. Normally, $\:{\upphi\:}$ = 111.13 km^36,51^.

## Results and discussion

### Temporal evolution trend of apple carbon emissions in China

#### Temporal characteristics of national apple carbon emissions

**Fig. 1:**
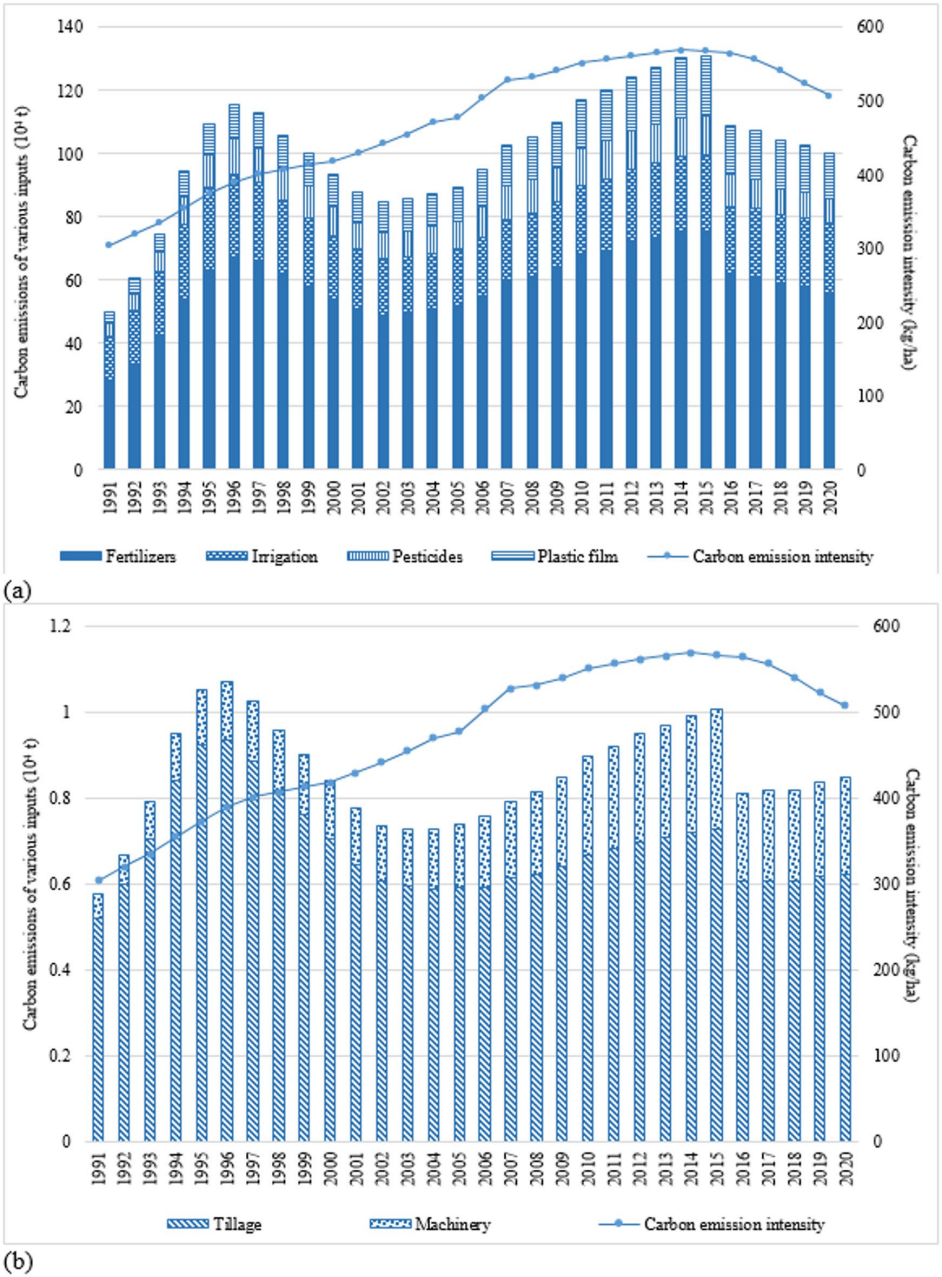
(a) and (b) Apple carbon emissions and intensity in China, 1991–2020.

Using the IPCC methodology for calculating carbon emissions, this paper measures China’s apple carbon emissions from 1991 to 2020 (Fig. 1(a) and (b)). In 2020, China’s apple carbon emission was 1.01 million tons, an increase of 100.38% from the base year of 1991 (0.51 million tons), with an average annual increase of 2.43%. Carbon emissions from plastic films, machinery, chemical fertilizers, pesticides, irrigation and tillage increased by 4.88, 4.76, 2.43, 2.14, 1.52 and 0.63% per year, respectively. Overall, apple carbon emissions showed an M-shaped fluctuation trend during the study period.

From its changes, China’s apple carbon emissions experienced the following four stages:

1. Rapid growth period (1991–1996): Due to the rapid growth of apple planting area into the late 1990s (increased from 1.66 million ha in 1991 to 2.99 million ha in 1996, with an average annual growth rate of 12.45%), farmers increased their input of chemical fertilizers, pesticides and other inputs to improve yields, leading to a sharp increase in apple carbon emissions from 0.51 million tons in 1991 to 1.16 million tons in 1996, with an average annual growth rate of 18.16%. Chemical fertilizers were the main driver of the rapid increase in apple carbon emissions during this period^52^. The introduction of a chemical fertilizers support policy in China, which provided for value-added tax (VAT) exemptions or rebates on some chemical fertilizers from 1994, resulted in the overuse of chemical fertilizers^9,29^. Therefore, carbon emissions from chemical fertilizers rapidly increased from 0.28 million tons in 1991 to 0.67 million tons in 1996, increased by 140.82% during six years.
2. Rapid decline period (1997–2002): apple carbon emissions decreased rapidly from 1.14 million tons in 1997 to 0.86 million tons in 2002, with an average annual reduction of 5.54%. Because of the restructuring of the apple industry and the excessive tax burden on agriculture during this period, a large number of low-yielding and inefficient orchards were eliminated across the country^32^. Apple planting area reduced rapidly from 2.84 million ha to 1.94 million ha, a reduction of 31.71%. This leads to a decrease in inputs, such as chemical fertilizers and pesticides, which in turn leads to a reduction in apple carbon emissions.
3. Slow growth period (2003–2015): influenced by technological advancements, improved logistical efficiency, and supportive policies (such as the abolition of agricultural taxes in 2006 and the establishment of a modern apple industry technology system in 2007), apple benefits continued to rise, motivating the majority of farmers to grow apples^34^. Apple planting area began to grow again, from 1.90 million ha in 2003 to 2.33 million ha in 2015, resulting in an increase in factor inputs, such as chemical fertilizers, pesticides and plastic film. Hence, apple carbon emissions increased gradually from 0.86 million tons to 1.32 million tons, with an average annual growth rate of 3.59%. Noteworthy, apple carbon emissions reached record peak in 2015.
4. Slow decline period (2016–2020): guided by environmental policies (such as apple double reduction project of chemical fertilizers and pesticides during the “Thirteenth Five-Year Plan” (2016–2020), stopping tax refunds and imposing a 13% VAT on chemical fertilizers in 2015), the amount of chemical fertilizers applied to apples reduced from 697.38 kilotons in 2016 to 624.96 kilotons in 2020, with a reduction of 10.39% ^29^. The amount of pesticides used to apples reduced from 20.28 kilotons to 15.63 kilotons, with a reduction of 22.93%. In addition, the amount of plastic film applied to apples reduced from 30.33 kilotons to 28.43 kilotons, with a reduction of 6.26%. As a result, apple carbon emissions started to decrease consistently, from 1.09 million tons to 1.01 million tons. While the layout of apple production also stabilized in this period, apple planting area was at around 1.93 million ha.

To sum up, although each carbon emission source increased by varying degrees, apple carbon emissions basically showed the same trend as apple planting area, which also indicates to some extent that the change in factor inputs resulting from the change in apple planting area is the main driver of changes in apple carbon emissions. Noteworthy, carbon emissions from chemical fertilizers are most evident.

This paper also uses apple carbon intensity to measure carbon emissions per unit area. China’s apple carbon intensity increased overall from 303.95 kg/ha in 1991 to 507.63 kg/ha in 2020, with an average annual growth rate of 1.78% (less than that of carbon emissions (2.43%)). This suggests that apple carbon emissions are increasing at a slower rate than apple planting area on a year-on-year basis. Unlike the change in carbon emissions, apple carbon intensity showed an inverted U-shaped trend. Especially after 2014, it started to decrease continuously, from 569.17 kg/ha in 2014 to 507.63 kg/ha in 2020. The main reason for the inconsistent trend in apple carbon emissions and intensity is the change in apple input factors, i.e., the shift from a single input of chemical fertilizers to a diversified input (such as pesticides, plastic films and machinery) with the improvement of apple technology and infrastructure.

**Fig. 2:**
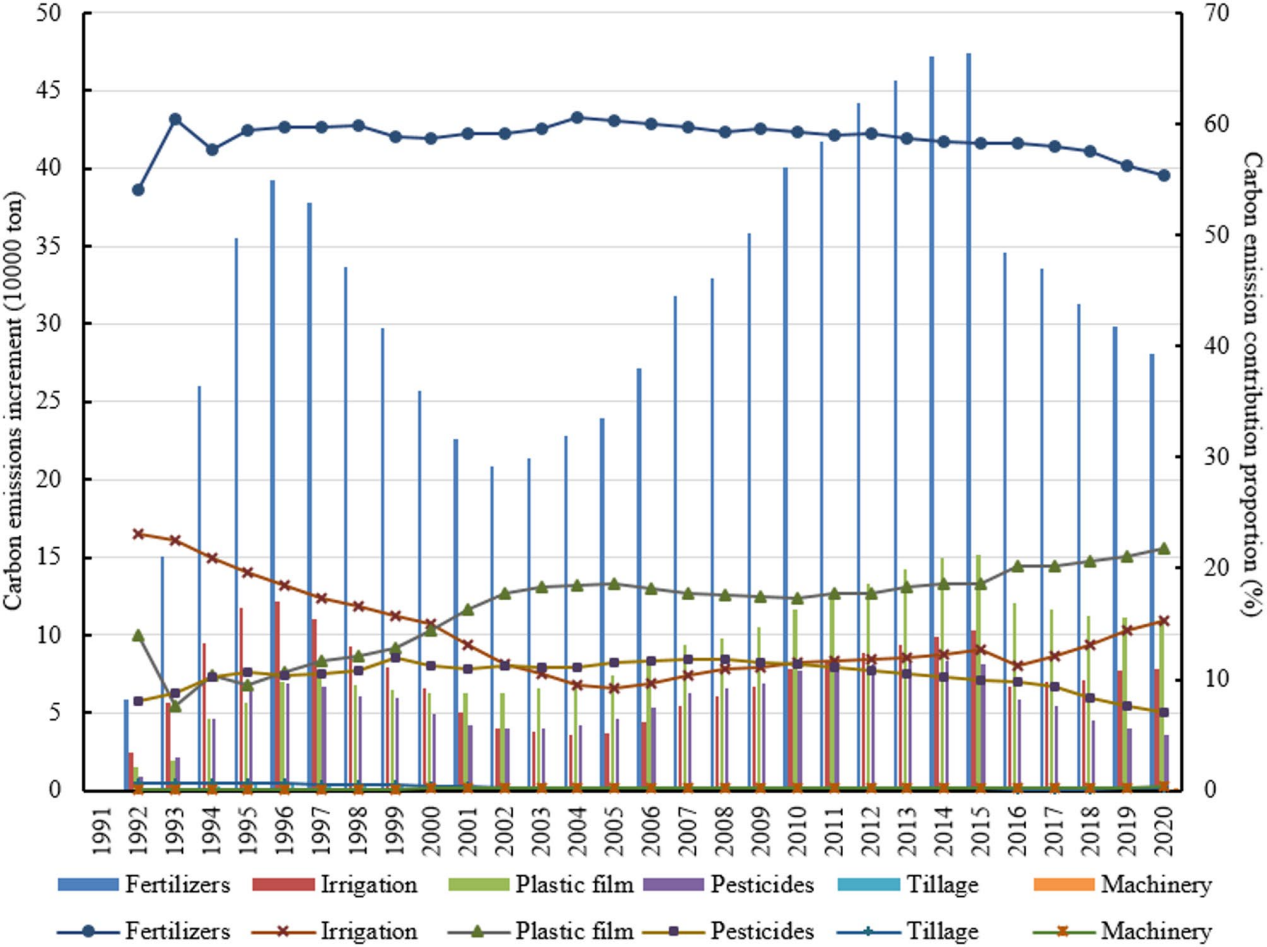
Increment and contribution proportion in China’s apple carbon emissions from each source. The bar chart corresponds to the left coordinate and the line chart to the right coordinate.

In terms of the increment and contribution proportion in apple carbon emissions from each source (Fig. 2), chemical fertilizers are the biggest contributor to the growth of apple carbon emissions, with its contribution increment increasing from 58.14 kilotons in 1992 to 280.68 kilotons in 2020, and its contribution proportion slightly increasing from 54.05 to 55.37%, which far superior to other carbon sources. Plastic film is the second largest driver for the growth of apple carbon emissions, with an average annual growth rate of 7.38 and 1.6% in its contribution increment and proportion, respectively. With the expansion of apple planting area in arid or semi-arid high-altitude regions, especially in the LPPA, the proportion of irrigable acreage decreased, resulting in the overall reduction of irrigation contribution to increment in apple carbon emissions. Its contribution proportion decreased from 23.07 to 15.35%, with an average annual reduction rate of 1.45%. Because of the increase in other inputs (such as chemical fertilizers and plastic films), pesticides contribution proportion was generally on the decline, from 8.08 to 6.99%. Due to the low level of mechanization and tillage in apple production, the contribution increment and proportion of both to carbon emissions was small. The contribution proportion of both was 0.33 and 0.20% in 2020, respectively.

#### Temporal characteristics of regional apple carbon emissions

**Fig. 3:**
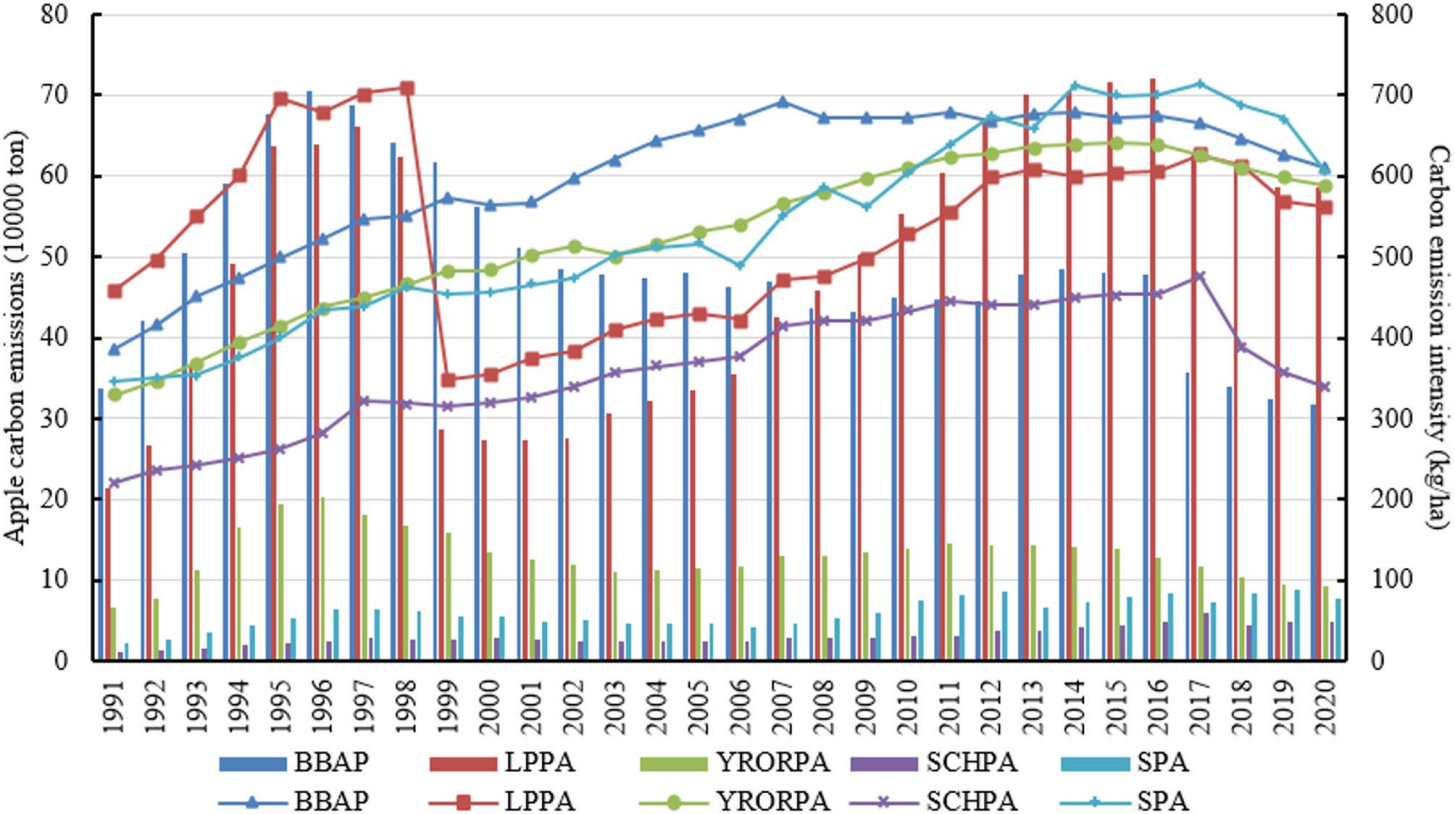
China’s apple carbon emissions and intensity in each production area. The bar chart corresponds to the left coordinate and the line chart to the right coordinate. BBPA denotes the Bohai Bay production area. LPPA represents the Loess Plateau production area. YRORPA refers to the Yellow River Old Road production area. SCHPA is the Southwest Cold and Highland production area. SPA represents the Special production area.

Apple carbon emissions showed significant regional heterogeneity across China’s five production regions (Fig. 3). In 1991, it showed BBAP > LPPA > YRORPA > SPA > SCHPA, in 2020 showed LPPA > BBAP > YRORPA > SPA > SCHPA, while in 1991–2020, on average, it showed BBAP > LPPA > YRORPA > SPA > SCHPA, indicating that in the major apple producing areas, especially apple carbon emissions are obviously high in the BBAP and LPPA, which implies that we need to focus on carbon emissions from these two regions. The BBAP and LPPA are the advantageous areas for apple production and the dominant areas for apple carbon emissions, these two areas accounted for 84.70% of national carbon emissions in 1991, and 80.46% in 2020. Noteworthy, with the expansion of apple planting area in the LPPA, it overtook the BBAP to become the largest region for apple carbon emissions in China after 2007, and Shaanxi is the biggest contributor to this shift^32^.

Similarly, the intensity of apple carbon emissions showed a large regional variability. In 1991, it showed LPPA > BBAP > SPA > YRORPA > SCHPA, in 2020 showed BBAP > SPA > YRORPA > LPPA > SCHPA, while in 1991–2020, on average, it showed BBAP > SPA > LPPA > YRORPA > SCHPA. Figure 3 reveals that the carbon intensity of the BBAP, SPA, YRORPA and LPPA, although fluctuating, is gradually converging, which all exceeded 560 kg/ha in 2020. Carbon emission intensity of the SCHPA, although showing an overall increasing trend, has been the lowest among the five production areas. These results indicate that although there is much variation in the carbon emission intensity across the five production areas, this variation is gradually decreasing and all are beginning to show a decreasing trend from 2017. China issued policies on the implementation of the five major actions for green agriculture development and other policies since 2017, which strengthened the guidance and regulation on the green and low-carbonization inputs for apple production materials^53^.

The contribution of each input to the growth of apple carbon emissions in each production area is illustrated in (Table 2). As the apple planting area of the LPPA continues to expand, the increase in chemical fertilizers, pesticides and other inputs in the LPPA contributed to the growth of its carbon emissions. From 1991 to 2020, the LPPA has the highest incremental carbon emissions at 372.16 kilotons, with an average annual growth rate of 3.53%. Chemical fertilizers, plastic films and irrigation are the main contributors, with the three contributing 347.14 kilotons or 93.28% of the increase in carbon emissions. Of this, chemical fertilizers contributed 52.41%. In contrast, the BBPA, also an advantageous area, reduced its carbon emissions by 20.59 kilotons, an average annual reduction rate of 0.22%. Because the BBPA is the earliest and highest production level of apple cultivation in China, its apple cultivation technology is more mature compared to other production areas, paying more attention to the environmental effects of apple production, the earliest use of soil testing and fertilizer recommendation, water and fertilizer integration and other energy-saving techniques. The carbon emission intensity of apples in the BBAP has been declining steadily since 2007, from 691.25 kg/ha in 2007 to 609.51 kg/ha in 2020. In addition, the growth in carbon emissions was modest in the SCHPA and SPA, but both had higher average annual growth rates of 5.05 and 4.39%, respectively. Fertilizer contribution proportion of these two production areas all closed to 50%, followed by plastic films and irrigation. The smallest change in apple carbon emissions is found in the YRORPA among the five production areas, its emissions merely increased from 65.73 kilotons in 1991 to 92.92 kilotons in 2020 by 27.19 kilotons.

Overall, except for the BBPA, apple carbon emissions increased to varying degrees in other four production areas, and the input contributions of these four production areas showed chemical fertilizers > plastic films > irrigation > pesticides > machinery > tillage, while showed plastic films > irrigation > chemical fertilizers > pesticides > tillage > machinery in the BBPA. It means that variations in apple carbon emissions are influenced by changes in apple production layout and technological progress^32,36^.

**Table 2:** Contributing factors to China’s Apple carbon increment by production area, 1991–2020.

Region	Increment^1^	Chemical fertilizers	Pesticides	Plastic film	Machinery	Irrigation	Tillage
BBAP	−2.06	−2.14	0.13	2.55	0.03	−22.53	−20.11
		(103.74)	(−6.16)	(−123.93)	(−1.68)	(122.63)	(5.39)
LPPA	37.22	19.50	2.23	8.94	0.09	6.27	0.18
		(52.41)	(6.00)	(24.02)	(0.24)	(16.85)	(0.48)
YRORPA	2.72	2.06	0.12	0.62	0.01	−0.08	−0.01
		(75.91)	(4.54)	(22.64)	(0.48)	(−3.10)	(−0.48)
SCHPA	3.75	1.85	0.24	0.87	0.01	0.75	0.03
		(49.22)	(6.52)	(23.14)	(0.28)	(20.08)	(0.76)
SPA	5.50	2.67	0.17	1.53	0.01	1.10	0.02
		(48.51)	(3.12)	(27.90)	(0.16)	(19.96)	(0.36)

1. Increment represents the increase in apple carbon emissions in each production area from 1991 to 2020, in 10 kilotons. The figures of columns 2 to 7 in the table showed the contribution increment of each input to the growth of apple carbon emissions in 10 kilotons in each production area, and the figures in brackets showed the contribution proportion in percentage.

#### Temporal characteristics of inter-provincial apple carbon emissions

**Fig. 4:**
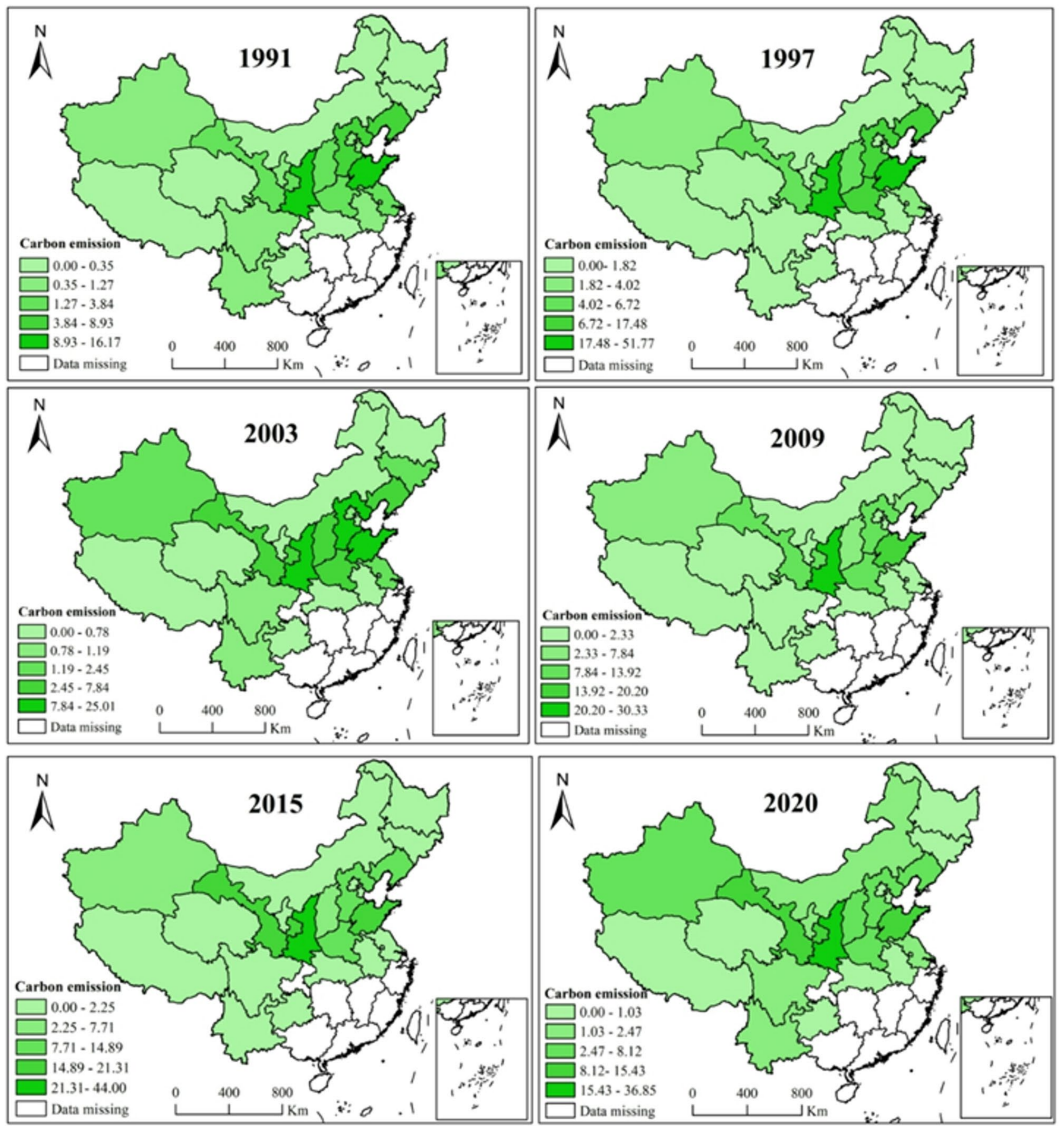
China’s apple carbon emissions for the years of 1991, 1997, 2003, 2009,2015 and 2020. (10 kilotons). This map is based on the standard map No. GS(2020)4632 downloaded from the standard map service website of National Administration of Surveying, Mapping and Geoinformation of China (http://bzdt.ch.mnr.gov.cn/) with no modification to the base map.

To understand the carbon reduction potential of apples in various production locations, it is essential to the spatial distribution of apple carbon emissions and develop differentiated carbon reduction techniques. Figure 4 showed the spatial distributions of apple carbon emission in 22 apple-producing provinces. Following the approach of Cui et al.^37^, six years (i.e., 1991, 1997, 2003, 2009, 2015 and 2020) out of 30 years were selected for this paper and the corresponding spatial distribution of apple carbon emissions for each year was plotted. It can be seen from Fig. 4 that the spatial distribution of apple carbon emissions has changed significantly over time. Particularly, in 1991, apple carbon emissions in Shandong, Shaanxi, Liaoning, Hebei, Henan, Shanxi and Gansu provinces exceeded 22 kilotons and average emission was 80.76 kilotons. In 1997, the average apple carbon emissions of these provinces increased rapidly to nearly 205 kilotons, increased by 153.49% during seven years. Noteworthy, apple carbon emissions in Shaanxi, Shandong and Hebei provinces increased rapidly from 1991 to 1997, exceeding 105 kilotons. However, apple carbon emissions decreased rapidly from 1997 to 2003, the average emission decreased from 73.76 kilotons to 43.91 kilotons as 40.47%. In 2003, apple carbon emissions started to grow again, the average apple carbon emissions grow to 52.56 kilotons by 2009. Noteworthy, apple carbon emission in Shaanxi, Shandong, Gansu, Hebei, Henan and Liaoning provinces exceeded 100 kilotons in 2015, and their average emission was 200 kilotons. In 2020, the average agricultural carbon emission of these provinces decreased to 146.05 kilotons, and Shaanxi and Hebei stood out in terms of carbon reduction with over 70 kilotons.

Overall, the spatial pattern of apple carbon emissions gradually changed from being dominated by Shandong, Shaanxi, Liaoning and Hebei (their emissions accounted for 73.41%) in 1991 to being dominated by Shaanxi, Shandong, Gansu, Liaoning, Hebei, Shanxi and Henan (their emissions accounted for 84.38%) in 2020. It reveals that the variance in apple carbon emissions among provinces is consistent with the variation in the key apple producing provinces.

The contribution of each input to the growth of apple carbon emissions in each province is shown in (Table 3). There are significant inter-provincial differences in apple carbon emissions and their contributing factors. From 1991 to 2020, thirteen provinces have all experienced varying degrees of growth in apple carbon emissions. Noteworthy, Shaanxi had the most significant increase in carbon emissions, up by 210.10 kilotons during the three decades, and fertilizer contribution is significant (contributed 62.99% of the carbon emission increment). Gansu increased by nearly 110 kilotons, and plastic films and chemical fertilizers contributed 43.43 and 31.42%, respectively. Shanxi and Xinjiang exceeded 40 kilotons, and chemical fertilizers contributed 54.22 and 48.20%, respectively. Henan increased by 30.60 kilotons, and fertilizer contribution is significant (contributed 75.43%). Inner Mongolia and Yunnan exceeded 13 kilotons, and fertilizer contribution is more significant (contributed 57.45 and 55.66%, respectively). The remaining six provinces (including Guizhou, Ningxia, Sichuan, Hebei, Heilongjiang, Tibet) had less than 10 kilotons of incremental carbon emissions.

Nine provinces reduced their apple carbon emissions. Noteworthy, Liaoning decreased by 8.10 kilotons, and fertilizers contribution is significant (contributed 74.91% of the carbon emission reduction). Shandong reduced by 7.36 kilotons, and fertilizers contribution is extremely significant (contributed 189.25%), but plastic films hinder carbon reduction by contributing up to 253.52%.

Overall, fertilizer contribution is more significant in thirteen provinces, particularly, its contribution proportion is extremely significant in Jilin and Tianjin, exceeding 80%. Plastic films in Hebei, Shandong and Beijing contributed more than 230% to carbon emission increment. Irrigation contribution in Hebei, Tibet and Heilongjiang is more significant.

**Table 3:** Contributing factors to China’s Apple carbon increment by Province, 1991–2020.

Region	Province	Increment^1^	Chemical fertilizers	Pesticides	Plastic film	Machinery	Irrigation	Tillage
BBAP	Shandong	−0.74	−1.39	0.17	1.87	0.02	−1.35	−0.05
			(189.25)	(−23.70)	(−253.52)	(−3.04)	(183.97)	(7.04)
	Hebei	0.23	0.46	−0.15	0.57	0.01	−0.65	−0.03
			(205.61)	(−65.28)	(253.53)	(4.33)	(−285.81)	(−12.37)
	Liaoning	−0.81	−0.61	0.16	0.00	0.00	−0.34	−0.03
			(74.91)	(−19.54)	(−0.27)	(−0.45)	(42.24)	(3.12)
	Tianjin	−0.68	−0.55	−0.01	−0.02	−0.00	−0.10	−0.00
			(80.36)	(2.06)	(3.24)	(0.07)	(13.95)	(0.32)
	Beijing	−0.06	−0.05	−0.04	0.13	−0.00	−0.09	−0.00
			(93.23)	(79.64)	(−237.71)	(1.40)	(156.53)	(6.90)
LPPA	Shaanxi	21.01	13.23	0.64	3.17	0.06	3.78	0.13
			(62.99)	(3.06)	(15.09)	(0.28)	(17.99)	(0.60)
	Gansu	10.97	3.45	1.18	4.77	0.02	1.52	0.04
			(31.42)	(10.75)	(43.43)	(0.21)	(13.82)	(0.38)
	Shanxi	4.53	2.46	0.41	0.82	0.01	0.82	0.01
			(54.22)	(9.01)	(18.20)	(0.14)	(18.18)	(0.26)
	Ningxia	0.86	0.49	0.01	0.18	0.00	0.18	0.00
			(56.54)	(1.18)	(20.52)	(0.22)	(21.31)	(0.23)
	Qinghai	−0.16	−0.12	−0.01	0.00	−0.00	−0.03	−0.00
			(75.81)	(4.84)	(−2.38)	(0.06)	(20.89)	(0.79)
YRORPA	Henan	3.06	2.31	0.20	0.45	0.01	0.10	−0.00
			(75.43)	(6.40)	(14.76)	(0.34)	(3.19)	(−0.14)
	Anhui	−0.05	−0.04	−0.00	0.04	0.00	−0.05	−0.00
			(73.88)	(2.41)	(−80.42)	(−1.67)	(98.35)	(7.44)
	Jiangsu	−0.29	−0.20	−0.07	0.12	0.00	−0.13	−0.00
			(71.20)	(24.73)	(−42.24)	(−0.58)	(45.20)	(1.70)
SCHPA	Sichuan	0.79	0.23	0.07	0.26	0.00	0.22	0.01
			(29.70)	(8.23)	(33.03)	(0.40)	(27.87)	(0.78)
	Yunnan	1.94	1.08	0.15	0.43	0.00	0.26	0.01
			(55.66)	(7.62)	(22.45)	(0.17)	(13.61)	(0.50)
	Guizhou	0.96	0.51	0.03	0.17	0.00	0.23	0.01
			(53.72)	(3.18)	(17.38)	(0.37)	(24.06)	(1.28)
	Tibet	0.07	0.02	0.00	0.01	0.00	0.04	0.00
			(29.51)	(2.44)	(8.71)	(0.92)	(57.90)	(0.53)
SPA	Inner Mongolia	1.35	0.78	0.05	0.22	0.00	0.30	0.01
			(57.45)	(3.48)	(15.94)	(0.22)	(22.25)	(0.65)
	Heilongjiang	0.12	0.03	0.01	0.00	0.00	0.07	−0.00
			(26.92)	(12.06)	(2.97)	(0.44)	(58.08)	(−0.46)
	Jilin	−0.05	−0.09	0.02	0.01	0.00	0.02	0.00
			(199.17)	(−37.33)	(−23.16)	(−1.16)	(−37.06)	(−0.46)
	Hubei	−0.17	−0.09	−0.02	−0.02	−0.00	−0.04	−0.00
			(54.54)	(9.77)	(8.86 )	(0.05)	(25.78)	(1.00)
	Xinjiang	4.26	2.05	0.11	1.32	0.00	0.76	0.01
			(48.20)	(2.57)	(31.02)	(0.11)	(17.79)	(0.30)

1. Increment represents the amount of increase in apple carbon emissions in each province from 1991 to 2020, in 10 kilotons. The figures of columns 2 to 7 in the table showed the contribution increment of each input to the growth of apple carbon emissions in 10 kilotons in each province, and the figures in brackets showed the contribution proportion in percentage.

### Spatial characteristics of apple carbon emissions in China

#### Spatial autocorrelation of apple carbon emissions

A simple comparison of the time-series data of apple carbon emissions in different periods can reveal the change trend of apple carbon emissions over time, but it cannot visualize the spatial distribution characteristics of apple carbon emissions in different periods. To compensate for the shortcomings of time-series analysis, this paper uses spatial autocorrelation to examine whether there is spatial autocorrelation between apple carbon emissions in each province and their surrounding provinces. As shown in Table 4, the Global Moran’s I index of apple carbon emissions is generally small, merely changing from −0.007 in 1991 to −0.015 in 2020, and all the Moran’s I values are insignificant. Except for 1999–2013, the value of the Global Moran’s I index was negative in the remaining years, and only in 1999–2001 was the value greater than 0.1. The above data suggest that there is no significant spatial correlation in China’s apple carbon emissions at the national level, and that they are mainly randomly distributed across the whole country, even with negative growth in individual years. In other words, apple carbon emissions from neighboring provinces showed a negative effect of “high-low” (H-L) clustering. In addition, the Global Moran’s I index decreases from −0.007 in 1991 to −0.015 in 2020, with an average annual rate of decline of 2.66%, and larger decreases appeared in some years.

**Table 4:** The global Moran’s I index of China’s Apple carbon emissions, 1991–2020.

year	Moran I	z	*p*-value^*^	year	Moran I	z	*p*-value^*^	year	Moran I	z	*p*-value^*^
1991	−0.007	0.317	0.751	2001	0.115	1.284	0.199	2011	0.011	0.481	0.630
1992	−0.017	0.243	0.808	2002	0.098	1.158	0.247	2012	0.000	0.412	0.680
1993	−0.011	0.298	0.765	2003	0.072	0.934	0.350	2013	0.001	0.414	0.679
1994	−0.001	0.387	0.699	2004	0.075	0.958	0.338	2014	−0.002	0.384	0.701
1995	−0.014	0.291	0.771	2005	0.075	0.953	0.341	2015	−0.006	0.359	0.720
1996	−0.008	0.329	0.742	2006	0.061	0.823	0.410	2016	−0.016	0.278	0.781
1997	−0.017	0.259	0.795	2007	0.058	0.793	0.428	2017	−0.013	0.316	0.752
1998	−0.020	0.239	0.811	2008	0.057	0.794	0.427	2018	−0.017	0.293	0.769
1999	0.129	1.451	0.147	2009	0.029	0.599	0.549	2019	−0.018	0.281	0.779
2000	0.115	1.297	0.195	2010	0.014	0.492	0.622	2020	−0.015	0.314	0.753

*Represents two-tail test.

Based on Eq. (5), this paper employs local spatial autocorrelation to verify whether apple carbon emissions have regional agglomeration characteristics, thus providing a graphic display of apple carbon emission trends in regional spatial variation. Followed the methods of Cui et al.^37^, this paper selects 6 years (i.e., 1991, 1997, 2003, 2009, 2015 and 2020) from 30 years, and mapped their Moran scatterplot of apple carbon emissions. As shown in Table 5, trends in the agglomeration of apple carbon emissions showed significant regional differences in China over time. In 1991, three provinces (including Shandong, Hebei and Henan) and ten provinces (including Beijing, Heilongjiang, Jiangsu, Anhui, Guizhou, Yunnan, Tibet, Gansu, Qinghai and Xinjiang) are located in the H-H and L-L spatial agglomeration regions, respectively, which indicates that Shandong, Hebei, Henan and their surrounding provinces all have high carbon emissions, ten provinces are surrounded by low-emission provinces. While there are seven provinces (including Tianjin, Shanxi, Inner Mongolia, Jilin, Hubei, Sichuan and Ningxia) in the L-H quadrant and two provinces (including Shaanxi and Liaoning) in the H-L quadrant. It is suggested that seven provinces with low apple carbon emissions have higher carbon emissions in their neighboring provinces, and two provinces with high apple carbon emissions have lower carbon emissions in their neighboring provinces. In 1997, the number of provinces in the L-L quadrant grew to eleven. In 2003, Shanxi and Shaanxi added to the H-H quadrant, and nine provinces in the L-L quadrant. When it comes to 2009, ten provinces in the L-L quadrant. In 2015, the number of provinces located in the L-L quadrant decreased to eight. Noteworthy, Hebei, Shanxi, Henan and Xinjiang in H-H quadrant and only seven provinces in the L-L quadrant in 2020. Overall, the number of provinces in the H-H and L-L quadrant decreased from thirteen in 1991 to eleven in 2020, which indicates that the structure of apple cultivation and the degree of technological progress vary greatly between provinces, resulting in significant inter-provincial differences in apple carbon emissions, with some provinces even experiencing a large shift from the H-H or L-L regions to the H-L or L-H regions in individual years^37,54^.

**Table 5:** Moran scatter distribution of China’s Apple carbon emissions for the years of 1991, 1997, 2003, 2009, 2015 and 2020.

Year	H-H	L-L	L-H	H-L
1991	Shandong, Hebei, Henan	Beijing, Heilongjiang, Jiangsu, Anhui, Guizhou, Yunnan, Tibet, Gansu, Qinghai, Xinjiang	Tianjin, Shanxi, Inner Mongolia, Jilin, Hubei, Sichuan, Ningxia	Shaanxi, Liaoning
1997	Shandong, Hebei, Henan, Shanxi	Beijing, Tianjin, Jilin, Heilongjiang, Jiangsu, Anhui, Guizhou, Yunnan, Tibet, Qinghai, Xinjiang	Inner Mongolia, Hubei, Sichuan, Ningxia	Shaanxi, Liaoning, Gansu
2003	Shandong, Shaanxi, Hebei, Henan, Shanxi	Beijing, Liaoning, Jilin, Heilongjiang, Jiangsu, Anhui, Guizhou, Yunnan, Tibet, Gansu, Xinjiang	Tianjin, Inner Mongolia, Hubei, Sichuan, Qinghai, Ningxia	Liaoning, Gansu
2009	Shaanxi, Shandong, Hebei, Henan, Shanxi	Beijing, Tianjin, Jilin, Heilongjiang, Jiangsu, Anhui, Guizhou, Yunnan, Tibet, Xinjiang	Qinghai, Ningxia, Inner Mongolia, Sichuan, Hubei	Liaoning, Gansu
2015	Shandong, Hebei, Henan, Shanxi, Xinjiang	Beijing, Tianjin, Jilin, Heilongjiang, Jiangsu, Anhui, Guizhou, Yunnan	Qinghai, Ningxia, Sichuan, Inner Mongolia, Hubei, Tibet	Liaoning, Gansu, Shaanxi
2020	Hebei, Shanxi, Henan, Xinjiang	Beijing, Tianjin, Jilin, Heilongjiang, Jiangsu, Anhui, Yunnan	Qinghai, Ningxia, Sichuan, Inner Mongolia, Hubei, Guizhou, Tibet	Shaanxi, Shandong, Gansu, Liaoning

#### Spatial barycenter of apple carbon emissions

**Fig. 5:**
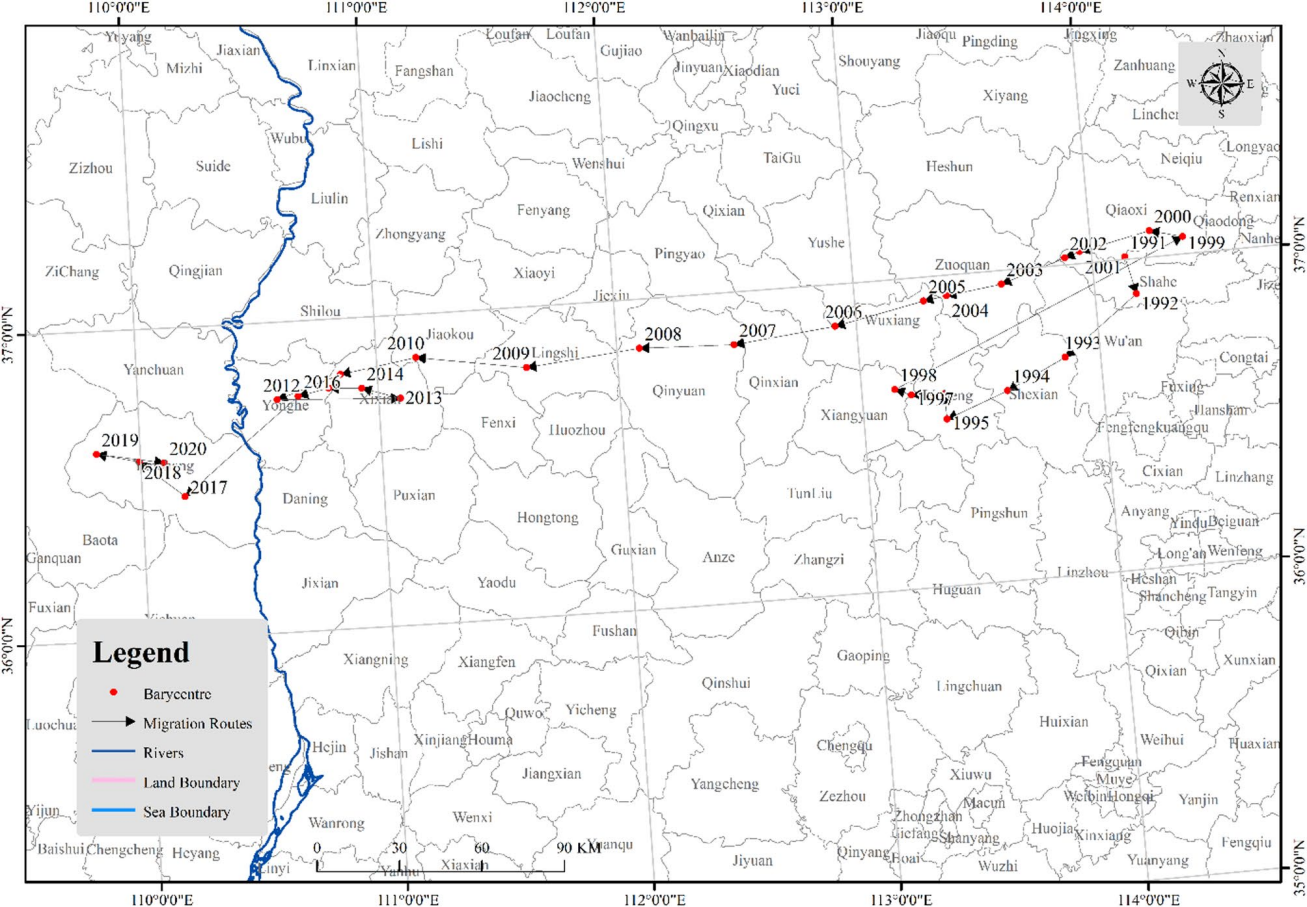
Spatial barycenter track of China’s apple carbon emissions. This map is based on the standard map No. GS(2020)4632 downloaded from the standard map service website of National Administration of Surveying, Mapping and Geoinformation of China (http://bzdt.ch.mnr.gov.cn/) with no modification to the base map.

The barycenter of apple carbon emissions portrays the equilibrium point of emissions in the overall spatial pattern, and its movement trajectory reflects the spatial transfer process over time, described by the direction and distance of its movement. As shown in Table 6; Fig. 5, the barycenter of apple carbon emissions moved southwestward by 451.69 km from 1991 to 2020, with notable displacements in certain years. In 1991, the center of apple carbon emissions was in Shahe City of Hebei Province. By 1995, it moved southwestward by 102.58 km and reached Licheng County of Shanxi Province. In 2000, it moved northeastward 117.56 km and returned to Qiaoxi District of Hebei Province. In 2017, the barycenter of apple carbon emissions continuously moved southwestward by 457.89 km, arriving in Yanchang County of Shaanxi Province. In 2020, it moved to northwest by 15.20 km and still located in Yanchang County of Shaanxi Province. The above changes align closely with the changes in apple planting area barycenter found by Zhang et al.^36^.

**Table 6:** Spatial barycenter diversion of China’s Apple carbon emissions.

Year	Longitude (°E)	Latitude (°*N*)	Direction	Distance (km)	Year	Longitude (°E)	Latitude (°*N*)	Direction	Distance (km)
1991	114.14	37.01	–	–	2006	112.91	36.87	Southwest	42.20
1992	114.17	36.89	Southeast	13.98	2007	112.49	36.83	Southwest	47.29
1993	113.85	36.70	Southwest	40.82	2008	112.09	36.84	Northwest	43.68
1994	113.60	36.61	Southwest	29.59	2009	111.62	36.80	Southwest	52.81
1995	113.34	36.54	Southwest	30.00	2010	111.16	36.86	Northwest	51.09
1996	113.34	36.62	Northeast	8.95	2011	110.85	36.82	Southwest	35.47
1997	113.20	36.62	Northwest	14.98	2012	110.58	36.75	Southwest	30.83
1998	113.14	36.65	Northwest	7.83	2013	111.09	36.73	Southeast	56.84
1999	114.39	37.06	Northeast	145.95	2014	110.93	36.77	Northwest	18.14
2000	114.25	37.08	Northwest	15.47	2015	110.79	36.78	Northwest	15.29
2001	113.95	37.04	Southwest	33.44	2016	110.67	36.76	Southwest	14.32
2002	113.89	37.02	Southwest	7.35	2017	110.18	36.46	Southwest	63.97
2003	113.62	36.96	Southwest	31.15	2018	109.99	36.57	Northwest	24.20
2004	113.38	36.93	Southwest	25.83	2019	109.82	36.61	Northwest	19.76
2005	113.29	36.92	Southwest	10.86	2020	110.10	36.57	Southeast	31.23

Direction and distance represent the moving direction and distance and of apple carbon emission barycenter in the current year relative to the previous year, respectively.

## Conclusions and policy recommendations

This paper uses the LMDI method, spatial autocorrelation analysis and barycenter analysis to describe the spatial and temporal dynamics of apple carbon emissions in China from 1991 to 2020. The main conclusions are as follows.

1. From the national dimension, apple carbon emissions underwent four stages (rapid growth period (1991–1996); rapid decline period (1997–2002); slow growth period (2003–2015); slow decline period (2016–2020)) Over this period, emissions increased 1.38-fold, while apple carbon intensity increased 67.01%. The average annual growth rate of apple carbon emissions (2.43%) was higher than that of carbon intensity (1.78%). Among the six primary sources of emissions, chemical fertilizers contributed the most, accounting for approximately 55% of the carbon increment, followed by plastic films, irrigation, pesticides, machinery, and tillage.
2. From the regional dimension, apple carbon emissions and their density presented significant regional heterogeneity during the study period. The spatial pattern changes from “LPPA > BBAP > SPA > YRORPA > SCHPA” in 1991 to “BBAP > SPA > YRORPA > LPPA > SCHPA” in 2020. Similarly, the spatial pattern of carbon intensity changed from “LPPA > BBAP > SPA > YRORPA > SCHPA” to “BBAP > SPA > YRORPA > LPPA > SCHPA”. Fertilizers contribute significantly to the growth of apple carbon emissions in LPPA, YRORPA, SCHPA and SPA, while the contribution of plastic films in BBAP is extremely significant.
3. From a provincial perspective, thirteen provinces showed varied trends in apple carbon emissions, with Shaanxi and Gansu exceeding 100 kilotons, whereas nine provinces lowered their apple carbon emissions, with Liaoning and Shandong cutting theirs by more than 7 kilotons. Fertilizers were the dominant contributors in thirteen provinces, plastic films in five provinces, and irrigation in three provinces.
4. Apple carbon emissions showed no significant spatial correlation between neighboring provinces and showed a negative effect of high-low or low-high clustering in individual years. The number of provinces in the H-L and L-H regions increased from 9 to 11. Traditional apple-producing provinces shifted from high-high agglomeration to low-low agglomeration, while emerging apple-producing provinces in central and western China transferred from low to high emissions. Meanwhile, the barycenter of apple carbon emissions moved southwestward by 451.69 km from Shahe City of Hebei Province to Yanchang County of Shaanxi Province.

In summary, apple carbon emissions showed a fluctuating growth trend, with a significant increase in apple carbon intensity. Fertilizers contributed the most carbon emissions among the six sources in most provinces. Chemical fertilizers production is heavily reliant on fossil energy sources, such as oil, and generates significant amounts of carbon emissions, whereas organic fertilizers, which are primarily derived from animal and plant residues, such as livestock manure and crop straw, can improve soil fertility and organic matter content while reducing carbon emissions^55^. Strategies such as organic fertilizer replacement^29–31^, water and fertilizer integration, and other fertilizer-saving techniques are essential to minimize apple carbon emissions.

Meanwhile, it is critical to focus on the role of plastic films and irrigation in the rise of apple carbon emissions, with both being reduced through mulch recycling, straw mulching, and drip irrigation. Region-specific emission reduction strategies should also be developed, considering their unique spatial and temporal characteristics of each region. Especially, greater attention should be paid to apple carbon emissions in the Loess Plateau production areas, such as Shaanxi and Gansu Provinces, and resource-saving technologies, such as organic fertilizer substitution and water-saving irrigation, should be adopted to reduce carbon emissions from apples in this region.

Admittedly, this paper may have the following limitations: First, the data on apple carbon emissions and their sources are indirectly estimated at the provincial level. Future studies could achieve greater accuracy by using municipal or county level data. Second, although the six main sources of apple carbon emissions are considered in this paper, there may be other inputs used in apple production that affect carbon emissions, such as insect traps and hail nets. Combining the characteristics of apple production with the construction of apple-specific carbon emission indicators and their conversion factors may enhance the reliability of the results. Finally, apple orchards have both negative and positive environmental footprint, as there is not only carbon emission, but also carbon sequestration during apple production, and carbon sequestration can be included to estimate the net apple carbon emissions in future studies.

## Data Availability

Data Availability Statement: All data generated or analysed during this paper are included in this published article.
